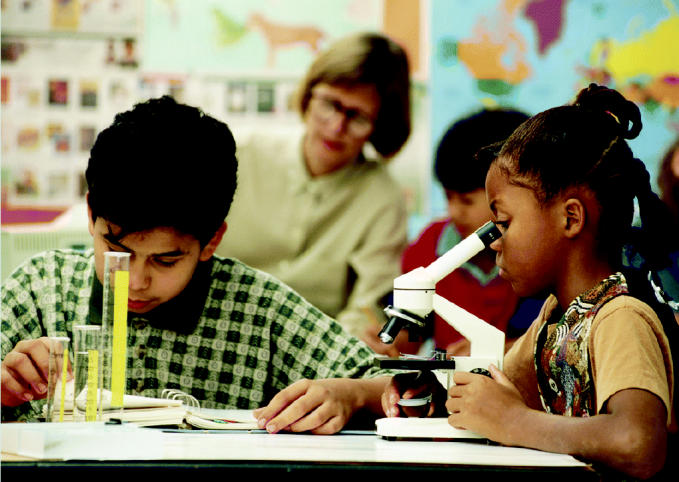# Science Education: The Future Begins Today!

**Published:** 2005-09

Today, more than ever, science education is essential for increasing our science literacy and cultivating the next generation of scientists. Science literacy is key to helping us make sense of the information we receive in this fast-paced world where technologic and scientific advances are made at an ever increasing rate. At a time when fewer U.S. students are pursuing careers in science and U.S. students are not faring as well in the sciences and mathematics as their counterparts around the world (Martin et al. 2004; National Center for Education Statistics 2004), science education can nurture student enthusiasm for science and ensure that the United States continues to contribute to scientific, technologic, and economic advances. Business and science leaders highlight that should these downward trends continue, the United States may experience adverse economic, social, and scientific consequences.

The NIEHS supports a kindergarten through 12th grade (K–12) science education grant program to increase student awareness of environmental health, stimulate student interest in science and academics, improve student academic performance, and enhance teachers’ ability to engage students. The NIEHS supports nine innovative education research projects to develop and disseminate inquiry-based curricula that use environmental health sciences as a central theme across different subjects (e.g., math, science, language arts, history, geography, civics). Key highlights of these projects demonstrate how the NIEHS science education program is working to prepare the next generation of scientifically literate and scientifically engaged citizenry.

Improved student achievement: Preliminary data from several projects indicate that their curricular materials improve students’ performance, especially for special-needs students. All projects report increased enthusiasm for science when students are exposed to the integrative curriculum.

Increased understanding of environmental health: Several projects report increased student understanding regarding the link between human health and the environment. In particular, students gained awareness of how science relates to their personal lives.

Social responsibility: Using a problem-based learning (PBL) curriculum, students learn from real-world experiences. They learn how to identify questions, conduct research, analyze data, and communicate recommendations. Projects using PBL show that students gain an appreciation about the link between science and social responsibility. Students are able to answer the question “How am I ever going to use this?”

Teacher participation: Teachers have limited time to learn and implement new curricula. However, several projects highlight how working closely with teachers from the very beginning to develop integrative environmental health curricula has led to greater buy-in from teachers and increased the use and sustainability of the materials.

For more information on the NIEHS science education program and the nine projects mentioned, visit **http://www.niehs.nih.gov/translat/k12/ehsic.htm.**

## Contact

**Liam O’Fallon** | ofallon@niehs.nih.gov

## Figures and Tables

**Figure:**